# The gut barrier as a gatekeeper in colorectal cancer treatment

**DOI:** 10.18632/oncotarget.28634

**Published:** 2024-08-14

**Authors:** Roy Hajjar, Carole Richard, Manuela M. Santos

**Affiliations:** ^1^Nutrition and Microbiome Laboratory, Centre de recherche du Centre hospitalier de l’Université de Montréal (CRCHUM), Montréal, Québec, Canada; ^2^Department of Surgery, Digestive Surgery Service, Centre hospitalier de l’Université de Montréal (CHUM), Montréal, Québec, Canada; ^3^Department of Surgery, Faculty of Medicine, Université de Montréal, Montréal, Québec, Canada; ^4^Institut du cancer de Montréal, Montréal, Québec, Canada; ^5^Division of General Surgery, Université de Montréal, Montréal, Québec, Canada; ^6^Department of Medicine, Faculty of Medicine, Université de Montréal, Montréal, Québec, Canada

**Keywords:** colorectal cancer, gut microbiome, gut barrier, colorectal surgery, anastomotic leak

## Abstract

Colorectal cancer (CRC) is highly prevalent and is a major cause of cancer-related deaths worldwide. The incidence rate of CRC remains alarmingly high despite screening measures. The main curative treatment for CRC is a surgical resection of the diseased bowel segment. Postoperative complications usually involve a weakened gut barrier and a dissemination of bacterial proinflammatory lipopolysaccharides. Herein we discuss how gut microbiota and microbial metabolites regulate basal inflammation levels in the gut and the healing process of the bowel after surgery. We further elaborate on the restoration of the gut barrier function in patients with CRC and how this potentially impacts the dissemination and implantation of CRC cells in extracolonic tissues, contributing therefore to worse survival after surgery.

## INTRODUCTION

Colorectal cancer (CRC) is among the most prevalent and deadliest cancers worldwide [1, 2]. Screening methods like fecal immunochemical tests (FIT) and colonoscopies allow for the early detection and excision of premalignant tumors – polyps – but the incidence of this cancer remains alarmingly high despite such measures [1–4].

The cornerstone of CRC treatment is surgery, in which the diseased segment of the bowel is resected and the remaining bowel ends are reconnected with an anastomosis. What used to be initially an invasive abdominal procedure has evolved into a minimally invasive operation in most patients using techniques like laparoscopy and robotic surgery [5]. Coupled with “enhanced recovery after surgery” (ERAS) protocols, these novel surgical techniques have decreased postoperative complications and allowed patients to recover more quickly after surgery [6, 7]. Nevertheless, improved surgical technique and perioperative care have eradicated neither postoperative complications nor cancer recurrence.

A surgical break of the bowel wall disrupts the gut barrier which undergoes repair in the days and weeks following surgery. This repair process is paramount to the prevention of postoperative infectious complications like anastomotic leak (AL) [8]. We and others have shown in the last few years that gut microbiota influences the healing process of the bowel and the restoration of the gut barrier after surgery [8–10]. Based on our recent work, we believe that weakened gut barrier function, namely due to poor healing after surgery, leads to persistent systemic low-grade inflammation and a higher risk of local and systemic cancer recurrence [8, 9, 11]. Improvement of the gut barrier function in patients with CRC is under the control of both macroscopic and microscopic factors.

## MACROSCOPIC FACTORS

The most severe form of a disrupted gut barrier is an overt leak of fecal material from the bowel, which usually occurs at the surgery site and specifically the anastomosis site. Colorectal AL is a major and unpredictable complication. It is expected to occur in approximately 3 to 19% of patients undergoing colorectal resection [12, 13]. This wide range depends on many factors, including the type of surgery being performed, technical factors and patient comorbidities [13–15]. These risk factors may be influenced by the gut microbiota to various degrees [10].

Macroscopically, there is consensus among surgeons that a gastrointestinal anastomosis should be free of tension and torsion [16, 17]. This premise could not be validated by epidemiological human studies due to ethical reasons, as an anastomosis cannot be mechanically left under tension or torsion after surgery. Nonetheless, it is logical to assume that mechanical stress could lead to anastomotic disruption simply by inducing rupture of the sutures or staple lines. It is common practice however to verify these mechanical aspects in the operating room, making their impact on the pathogenesis of AL probably low.

## MICROSCOPIC FACTORS

### Epithelial proliferation

The inner layer of the colonic wall is in contact with the microbial communities in the lumen. It is estimated that 70% of the bacteria in the body reside in the colon and rectum [18]. It is therefore safe to assume that the healing process after an invasive surgical procedure would be influenced by the abundant gut microbiota in the bowel lumen and would be dependent on the pathogenic potential and biological functions of the bacteria involved [8, 9].

The healing of the epithelial layer is a frequently assessed parameter in animal models of bowel injury [19, 20]. Epithelial healing is usually studied in conditions where the epithelial lining is the most affected, as is the case for inflammatory bowel disease (IBD) and other inflammatory conditions. In a surgical context, even if the epithelial layer is not what provides tensile strength to the anastomosis, it constitutes an important barrier against gut pathogens, and its sealing may be expected to protect the deeper submucosal matrix against deleterious inflammatory stimuli [21].

In germ-free mice, the inoculation with a complex microbiota induces maturation of the epithelium along with increased proliferation [22]. In our previous work, we have shown for the first time that healing after colonic surgery is severely impaired in germ-free mice [8]. Epithelial regeneration requires nutritional substrates, and more specifically microbiota-derived substrates. Short-chain fatty acids (SCFAs) are among the major nutritional substrates for the gut epithelial lining [23]. They are known to promote epithelial integrity and gut barrier function [24, 25]. SCFAs are bacterial metabolites that are produced by colonic bacteria upon the fermentation of dietary fibers [26]. These fibers escape digestion in the small bowel and are therefore metabolized mostly by bacteria in the cecum and the proximal colon, a process that culminates in the production of SCFAs [27]. Butyrate, propionate and acetate are the main SCFAs produced in the large bowel [26].

Among these metabolites, butyrate is a major energy source for epithelial cells [28]. It promotes the proliferation of colonocytes and is expected to contribute therefore to the regeneration of the injured mucosa [23, 29]. In addition to promoting epithelial proliferation, butyrate was shown to enhance the gut barrier function by promoting the development of interepithelial tight junctions [30, 31]. The reinforcement of the gut barrier function by butyrate was also mediated by an enhancement of the mucus layer, specifically by an upregulation of MUC-2, the major mucin produced by epithelial cells [32]. These beneficial properties protect against systemic invasion by luminal pathogens.

This key bacterial metabolite is therefore expected to be of potential interest in colorectal surgery. The effects of butyrate have been assessed in the context of colonic anastomotic healing in murine models [33–35]. These experiments suggested that this SCFA improves anastomotic healing and the tensile strength of the anastomosis, but the findings were inconsistent [33–35]. From a practical perspective, oral administration of butyrate after surgery would expose the gut to this metabolite for only a short period of time, without considering the significant distance that separates the mouth from the colon and rectum. Another option would be intrarectal administration, but this approach might not be well received by the surgical community, as enemas may pose an undesirable mechanical stress on a fresh and fragile colorectal anastomosis. The alternative in this case would be to promote the production of this metabolite by the gut microbiota to consistently increase its luminal levels in the gut, in addition to ensuring a continuous mucosal exposure.

We previously conducted animal experiments in which we exploited the benefits of butyrate and other SCFAs in the healing of colonic anastomoses in mice [21]. Due to the limitations of direct administration of butyrate, our strategy was to modulate the colonic microbiota toward a butyrogenic profile that produces higher levels of SCFAs. The approach we tested was a supplementation with fermentable fibers, specifically oligosaccharides. Mice therefore received dietary supplementation with inulin or galacto-oligosaccharides (GOS) for 2 weeks before undergoing a colonic anastomosis under general anesthesia. The supplementation continued after surgery until sacrifice on postoperative day (POD) 6. Mice supplemented with inulin and GOS displayed increased levels of SCFAs in the gut and improved macroscopic healing of the anastomosis [21]. With respect to what was mentioned above, our experiment showed that the increased levels of butyrate, propionate and acetate coincided with an improved microscopic healing of the anastomosis. When assessed by a pathologist blinded to the intervention group, the wound site in mice supplemented with oligosaccharides was found to exhibit higher reepithelization and continuity of the mucosal layer. Since butyrate is known to promote epithelial proliferation, we assessed this parameter by quantifying the Ki-67 proliferation marker at the wound site by immunohistochemistry. Interestingly, this marker was found to be increased in mice supplemented with the inulin prebiotic. These findings support the role of these SCFAs, and of the microbiota, in the healing of the colonic anastomosis and restoration of the epithelial layer and therefore of the gut barrier after a surgical injury.

Considering the above, it is important to assess the effect of microbiota-mediated epithelial proliferation in the specific context of cancer. Patients with CRC constitute a large proportion of patients undergoing colorectal resections with anastomosis. It is therefore important to ensure that higher bacterial-derived butyrate levels will not increase the proliferation of cancer cells and promote cancer progression. This consideration is valid, but the short period of supplementation with such oligosaccharides will most probably not affect cancer progression and the overall oncological prognosis. Most importantly, while butyrate is known to promote the proliferation of normal colonocytes, it is known to inhibit that of cancer cells, a process that is best known as the “butyrate paradox” [36]. We have previously published a review article on this specific and yet complex question [23]. Briefly, in physiological circumstances, colonocytes metabolize butyrate via mitochondrial oxidation [23, 37]. In neoplastic cells, specifically colorectal adenocarcinoma cells, the cellular metabolism shifts toward aerobic glycolysis, which induces an accumulation of unused butyrate [23, 36, 38]. Butyrate exerts a histone deacetylase (HDAC) inhibitor function, which prevents the modulation of chromatin toward a pro-carcinogenic profile by inhibiting proto-oncogenes and activating tumor suppressor genes [23, 39–41]. In short, in patients with CRC, targeting the microbiota to improve epithelial proliferation through SCFAs may be beneficial not only against anastomotic complications but also against cancer progression.

### Submucosal recovery

The submucosa is believed to confer tensile strength and solidity to the anastomosis, as it harbors a high concentration of connective tissue and collagen [14, 21]. It may be considered therefore as the cornerstone of anastomotic healing. This layer is not frequently assessed in injury models as these usually concentrate on mucosal injuries and do not routinely involve a radical transection of the colonic wall. The overwhelming body of evidence on the relation between the gut microbiota and anastomotic leak focuses on the preservation of collagen content at the anastomotic site.

Many bacterial species have been shown to activate colonic collagenases. Among these, the most famous one is *Enterococcus faecalis* [42]. *E. faecalis* is a Gram-positive commensal that is often incriminated in cases of nosocomial infections, including urinary tract infections, wound infections, endocarditis and bacteremia [43]. The team of Dr. John Alverdy at the University of Chicago has shown that some strains of *E. faecalis* have the capacity to activate matrix metalloproteinase 9 (MMP-9), a collagenolytic enzyme, which induces a degradation of anastomotic collagen and AL [42, 44]. Other bacterial species have been shown by the same team to activate intestinal collagenases as well, contributing to the pathophysiology of AL. Among these, *Pseudomonas aeruginosa*, *Serratia marcescens* and *Bacillus subtilis* were shown to induce a degradation of the extracellular matrix at the anastomotic site, thus preventing proper wound healing [45, 46]. Collagenase-producing bacteria are not present in all patients with anastomotic complications [47], suggesting that the healing process and the implication of the microbiota are more complex and involve other factors.

In our previous work, we have shown that fecal microbiota transplantation (FMT) using preoperative fecal samples from patients with AL induced poor anastomotic healing in mice when compared to FMT from patients with optimal healing [8, 9]. In the mice transplanted with feces from leaky patients, the anastomosis was shown to harbor lower collagen concentrations, as assessed by the concentration of hydroxyproline. Higher collagenase activity was identified as well in these mice, but the incriminated collagenolytic enzyme was MMP-2 with little to no involvement of MMP-9 [8, 21]. This sheds light on the complexity of the microbiota effect on colonic healing after surgery, and the necessity to determine reliable biomarkers to properly assess the risk of leak before surgery. We specifically showed that strains of *Parabacteroides goldsteinii* and *Alistipes onderdonkii* resist the preoperative bowel preparation and directly modulate the inflammatory reaction in the bowel mucosa after surgery, leading sometimes to destructive rather than reparative inflammation [8].

Bacterial metabolites like SCFAs may preserve the collagen layer and strengthen the submucosa after a surgical injury. In our experiment with oligosaccharides [21], mice fed the inulin and GOS-supplemented diets were shown to harbor higher collagen content at the anastomotic site. This was thought to be due to the ability of butyrate to inhibit collagenase expression. Previous work has suggested that butyrate can inhibit matrix metalloproteinases in joint cartilage [48], but this effect was not clearly demonstrated in the gut. Our data showed that butyrate, but also propionate and acetate, were associated with higher levels of hydroxyproline at the anastomosis, and with lower levels of collagenolytic activity at the wound site as well [21].

### Ischemia and epithelial oxygenation

The perfusion of colorectal anastomoses is considered a vital factor in the healing process after surgery, as blood supply provides the necessary oxygenation, nutrients and molecules required to maintain tissue viability and to promote repair mechanisms [49, 50]. This led to the implementation in surgical practice of strategies to assess colonic perfusion when this parameter is suspected to be compromised [51, 52]. Suboptimal perfusion of the anastomosis has been further shown to impair the healing process after surgery in mice [53], reinforcing the importance of this factor in the pathogenesis of anastomotic leak in surgical practice.

Perfusion is believed to be directly related to the integrity of the vessels that irrigate the anastomosed bowel segment. This integrity could be due to technical operative factors such as the level of ligation, or to the health of the patient’s vessels, namely in the case of chronic vascular disease [54]. There is no evidence of a potential role of the gut microbiota in these rather mechanical factors. Besides, there is no indication of a potential mechanism by which gut bacteria may influence vascular supply. However, gut bacteria may modulate epithelial oxygenation microscopically [55, 56].

Higher epithelial oxygenation was linked to colonic inflammation, carcinogenesis, and the expansion of deleterious species like *Escherichia coli* [57]. The maintenance of a hypoxic environment was reported to promote crosstalk between the microbiota and the host, nutrient absorption, and the maintenance of the barrier function [55, 58]. These beneficial effects may be mediated by the hypoxia-induced factor (HIF) [59]. In the context of colonic anastomoses, healing seems to be improved by hyperoxia according to a systematic review, and this improvement was inversely correlated with the abundance of gut anaerobes, which were reported to be increased by hypoxia and associated with poor healing [60]. The findings of these different reports are not in agreement, especially given that anaerobes are reported in other manuscripts as being beneficial for gut homeostasis [61, 62]. This warrants further work on how hypoxia and hyperoxia interact with the microbiota and influence the gut barrier function, and how this balance regulates healing in the specific context of invasive colorectal surgery.

### Inflammatory stimuli

The major mechanism that our previous work unveiled is that microbiota-mediated low-grade inflammatory signals directly affect repair mechanisms and the restoration of the gut barrier after surgery [8, 9]. In the wound healing process, there is an initial inflammatory phase characterized by the infiltration of polymorphonuclears (PMNs) at the injury site and the release of pro-inflammatory cytokines and chemokines including tumor necrosis factor alpha (TNF-α), interleukin-1 beta (IL-1β), IL-6 and monocyte chemoattractant protein 1 (MCP-1) [63]. During this repair process, there is a polarization of macrophages from the pro-inflammatory M1 to an M2 phenotype, which induces an anti-inflammatory response to promote regeneration and healing [63, 64]. During this transition, TNF-α decreases and the production of transforming growth factor beta (TGF-β) increases [65, 66]. This cytokine promotes fibroblastic activity and the accumulation of components of the extracellular matrix at the wound site, including collagen and fibronectin [67, 68]. In this phase, M1 macrophages transition toward an M2 phenotype, which promotes regeneration and remodeling of the wound site [69–71]. When the transition from the inflammatory phase to the proliferation phase is dysregulated, excessive inflammatory signals persist and prevent the wound from healing properly [69, 72]. Impaired healing leads to chronic wounds, which drive chronic inflammation, and which promotes the growth of bacteria at the wound site that become resistant to local bactericidal processes [69, 72, 73]. Bacterial growth at the wound site drives local inflammation, leading to a vicious circle where the continuous production of reactive oxygen species (ROS) and cytokines leads to the production of destructive enzymes and sustained inflammation, which prevents the healing process from evolving [74, 75]. TNF-α is believed to be an important driver of chronic inflammation in chronic non-healing wounds [76], and its suppression with anti-TNF agents was shown to help resume the normal healing process [77, 78].

This dysregulation and polarization toward a pro-inflammatory profile is also a hallmark of chronic intestinal inflammation, namely Crohn’s disease and ulcerative colitis [79–81]. Inflammation is known to correlate with the activation of MMPs, and inflammatory cytokines are believed to be activators of these collagenases [82–84]. Environmental factors that contribute to a pro-inflammatory state are therefore expected to prevent wound healing and to generate a chronic injury in the bowel, therefore weakening the gut barrier.

Gut bacteria are potent regulators of intestinal inflammation. Several species are known to activate the inflammatory cascade while others may harbor anti-inflammatory properties. Among pro-inflammatory microbes, members of the Enterobacteriaceae family have been shown to induce intestinal inflammation, including *Escherichia coli*, *Salmonella*, *Citrobacter rodentium* [85], and others like *Helicobacter hepaticus* [86, 87]. Gut bacteria may also have anti-inflammatory properties that can be mediated by beneficial metabolites such as the SCFAs presented earlier [88, 89]. Other species may induce an anti-inflammatory response either by inhibiting the production of pro-inflammatory cytokines or by promoting the production of anti-inflammatory molecules [90]. These bacteria include *Bifidobacteria*, *Lactobacilli*, *Bacteroides spp.* and *Parabacteroides spp.* [90–92]. By modulating the inflammatory response, gut bacteria may exacerbate or alleviate acute inflammation at the anastomotic site and consequently modulate the healing process of the gut barrier. In murine models of colonic anastomosis, the modulation of peritoneal inflammation was shown to influence the healing at the surgical site, and the isolation of the anastomosis in rats with peritonitis was further shown to prevent the deleterious effects of excessive peritoneal inflammation on anastomotic healing [93, 94].

In our work, we found that a subclinical pro-inflammatory state in the colonic mucosa of patients with CRC leads to poor restoration of the gut barrier after surgery [8, 9]. This pro-inflammatory environment was shown to be mediated by the gut microbiota, as the transfer of patient microbiota to mice induces the same increase in many pro-inflammatory cytokines in the mice colonic tissue [8, 9]. We found that several bacterial strains modulate the levels of inflammatory cytokines both in the mucosa and in the intraluminal environment, leading sometimes to a heightened level of systemic inflammation before surgery as demonstrated by higher circulating levels of white blood cells (WBCs) [8, 9]. We also showed that mild suppression of WBCs systemically, specifically monocytes and neutrophils, improved anastomotic healing and prevented bacterial translocation, suggesting that a controlled rather than a chaotic inflammatory reaction after surgery is highly beneficial to restore the gut barrier [8].

Interestingly, we found that the modulation of local and systemic inflammation by specific bacterial strains, specifically *P. goldsteinii*, was modulated by the peroxisome proliferator-activated receptor-gamma (PPAR-γ), which is activated by butyrate [11]. This pathway is particularly of interest in CRC as it reinforces the gut barrier and may further inhibit cancer cell proliferation and tumor progression [23]. Its activation by gut microbiota or bacterial metabolites not only improves surgical healing but also promotes favorable oncological outcomes [11].

## GUT BARRIER RECOVERY AND COLORECTAL CANCER

In our most recent work, we shed light on how the consolidation of the gut barrier using prebiotics prevents not only local but also systemic dissemination of cancer cells, influencing therefore disease-free and overall survival [11].

We found in this work using long-term clinical data that patients with poor postoperative healing and AL experience more local and distant cancer recurrence, and lower overall survival [11]. Based on these findings, we aimed at evaluating whether a weaker gut barrier at the bowel anastomosis site may allow residual cancer cells to escape the bowel lumen, and found that this was the case. We therefore tested whether PPAR-γ-stimulating compounds may prevent cancer cell proliferation, dissemination and implantation in extracolonic tissue. We assessed dietary supplementation with inulin, which promotes butyrate production, and 5-aminosalicylate (5-ASA), a direct PPAR-γ activator. We found that both supplements reinforced the gut barrier function, prevented the escape of cancer cells and diminished tumor burden [11].

Most importantly, we found that a stronger gut barrier prevented the escape of enteric bacteria out of the gut, postoperative sepsis, and the translocation of cancer-promoting bacterial lipopolysaccharides (LPS) into the circulation [11]. This led to a less proinflammatory and potentially less procarcinogenic systemic environment and was associated with a lower fixation and proliferation of cancer cells in the liver [11].

## FUTURE DIRECTIONS

**Figure 1:**
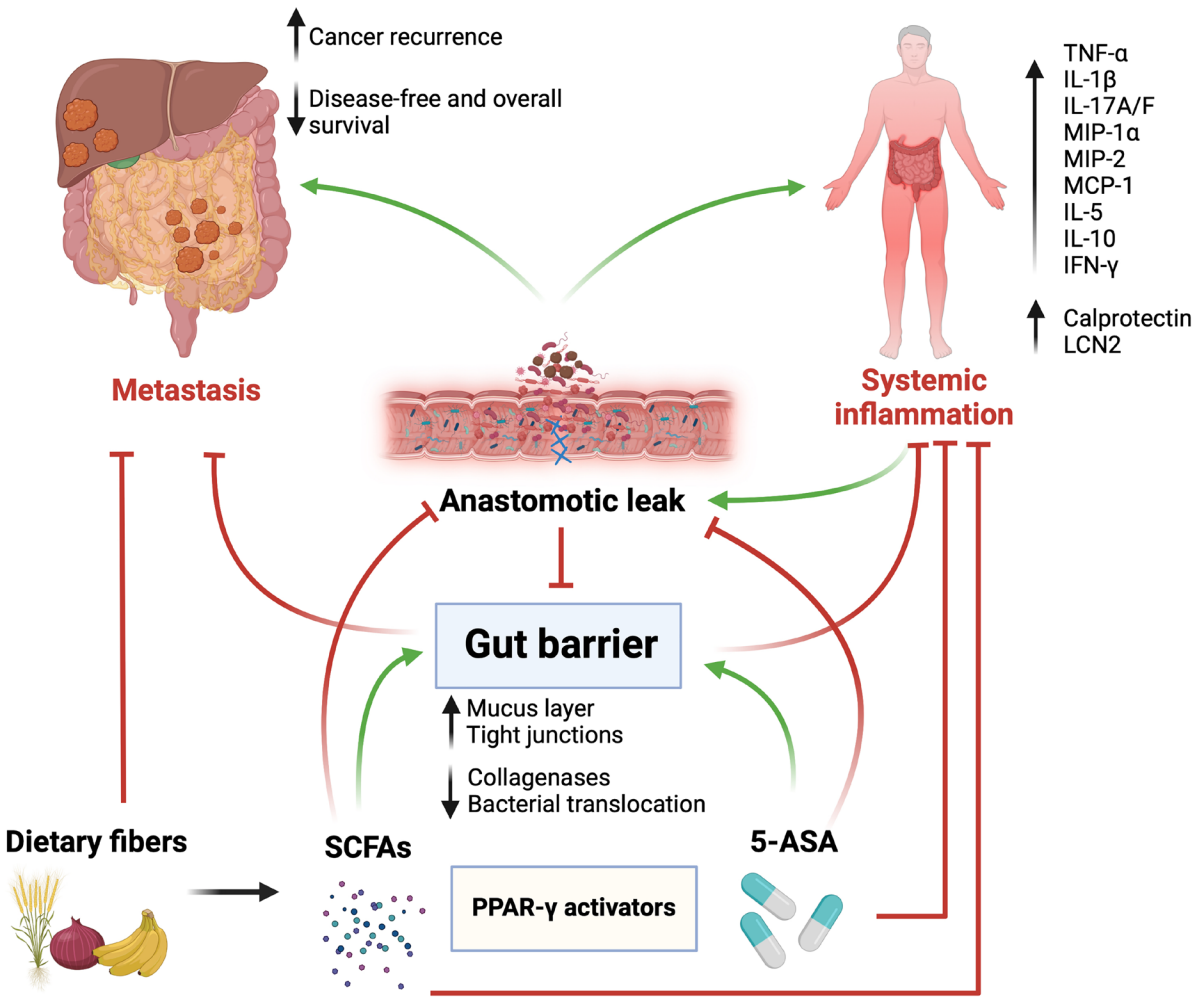
Graphical abstract. Surgery and anastomotic leak (AL) weaken the gut barrier function, which promotes the dissemination and implantation of colorectal cancer cells in the peritoneal cavity and distant organs. A disrupted gut barrier leads to intestinal and systemic inflammation that promote carcinogenesis and decrease survival. Activation of peroxisome proliferator-activated receptor-gamma (PPAR-γ) by 5-aminosalicylate (5-ASA) and short-chain fatty acids, derived from bacterial fermentation of dietary fibers, reinforces the gut barrier, prevents AL, decreases inflammation, and ultimately improves oncological outcomes. Abbreviations: TNF-α: tumor necrosis factor alpha; IL1β: interleukin 1 beta; IL17A/F: interleukin 17 A/F; MIP-1α: macrophage inflammatory protein 1 alpha; MIP-2: macrophage inflammatory protein 2; MCP-1: monocyte chemoattractant protein 1; IL-5: interleukin 5; IL-10: interleukin 10; IFN-γ: interferon gamma; LCN2: lipocalin 2. Green arrows indicate promotion/stimulation, red arrows indicate inhibition/prevention. Created with https://www.biorender.com/.

These findings pave the way toward dietary supplements that may stimulate PPAR-γ in an effort to consolidate the gut barrier function and prevent the systemic spread of procarcinogenic factors through a weakened colorectal mucosal layer. Such supplements may include fermentable dietary fibers and 5-ASA. The latter is a commonly used medication in patients with inflammatory bowel disease, and may therefore act as an anti-inflammatory agent to alleviate low-grade inflammation in patients at risk of experiencing poor anastomotic healing [95]. Other anti-inflammatory agents include biological ones, namely anti-TNF-α monoclonal antibodies [96, 97]. While this approach does not necessarily affect PPAR-γ, it was shown in our previous work to improve anastomotic healing in mice [8]. Nonetheless, such agents harbor non-negligible side effect profiles and costs [98–100]. Furthermore, even if excessive TNF-α may be deleterious in the tissue repair process, it seems to drive some vital functions in tissue remodeling. These include the activation of the inflammatory response at the wound site, and the subsequent modulation of cytoskeleton elements and keratinocytes that promote regeneration and tissue repair [101] (Figure 1).

Finally, reinforcing the gut barrier may alleviate systemic inflammation in patients with CRC and prevent the emergence of an oncogenic environment both locally in the bowel and systemically. This may in turn prevent the escape of cancer cells and their implantation in the peritoneal cavity or in distant organs. Ultimately, we showed that gut barrier integrity not only protects against postoperative sepsis and surgical complications, but is a pivotal factor in the prevention of local and systemic cancer recurrence, and perhaps in the response to systemic therapy. Future clinical studies are now urgently required to assess whether barrier-reinforcing agents improve outcomes in patients with CRC.
